# Global research trends in stroke and gut microbiota: a multi-database bibliometric analysis and cross-validation study

**DOI:** 10.3389/fmicb.2026.1766228

**Published:** 2026-04-08

**Authors:** Yawen Wang, Zhen Qiao, Jiacheng Zheng, Dan Wang, Wulin Gao, Maoxia Fan

**Affiliations:** 1 Dongzhimen Hospital, Beijing University of Chinese Medicine, Beijing, China; 2 Beijing University of Chinese Medicine, Beijing, China; 3 Heze Traditional Chinese Medicine Hospital, Heze, Shandong Province, China; 4 Wangjing Hospital of China Academy of Chinese Medical Sciences, Beijing, China; 5 Affiliated Hospital of Shandong University of Traditional Chinese Medicine, Jinan, Shandong Province, China

**Keywords:** bibliometrics, gut microbiota, hotspots, research trends, stroke

## Abstract

**Background:**

The fields of stroke and the gut microbiota are closely linked via the “gut-brain axis,” and their complex bidirectional interactions have emerged as a significant research focus. This study represents the first systematic bibliometric analysis of this field, aiming to delineate its knowledge structure, evolutionary trajectory, current research hotspots, and emerging frontiers.

**Methods:**

Data for this study were retrieved from the Web of Science Core Collection, covering the period from January 1, 2000, to August 31, 2025. Following a screening process, a total of 1,236 relevant articles were included in the analysis. Bibliometric and visualization tools, including CiteSpace, VOSviewer, R software, and Microsoft Excel, were employed to systematically analyze the distribution of research disciplines, publication output, contributions by country/region, institutional collaborations, influential authors, core journals, co-cited references, and keyword co-occurrence. Furthermore, to assess the robustness of our findings and explore characteristic differences across databases, we performed a multi-database validation and comparative analysis using data from Scopus and PubMed.

**Results:**

The number of annual publications has shown a continuous increase, with a rapid surge in the number of articles published over the past 5 years, achieving an average annual growth rate of 20.6%. China leads the world in terms of the number of publications, while the United States plays a central role in early-stage research and international collaboration. This study demonstrates significant interdisciplinary integration, encompassing multiple disciplines including neuroscience, pharmacology, immunology, and microbiology. Current research hotspots focus on the interaction between gut microbiota dysbiosis and post-stroke neuroinflammation, the therapeutic potential of microbial metabolites (e.g., short-chain fatty acids), the exploration of gut-brain axis mechanisms, and probiotic intervention strategies. Mechanistic research and clinical translation have been identified as the primary drivers for the development of this field. Multi-database validation showed that the annual publication trends, keyword distribution, and rankings of major contributing countries were consistent.

**Conclusion:**

Current research hotspots have expanded from basic mechanisms toward clinical translation, underscoring the importance of elucidating common pathophysiological mechanisms and identifying potential therapeutic targets. Future research priorities include refining screening and diagnostic protocols for gut microbial biomarkers, developing effective prevention strategies based on probiotics and other microbiota-targeted interventions, and advancing individualized and targeted therapeutic approaches. By integrating multi-omics data with precision medicine approaches, the field is poised to further accelerate the translation of mechanistic discoveries into clinical practice, ultimately offering novel strategies for the prevention and treatment of stroke.

## 1 Instruction

Stroke is an acute cerebrovascular disease resulting from the sudden rupture or occlusion of cerebral blood vessels, leading to focal brain tissue damage. It is characterized by high incidence, disability, mortality, and recurrence rates (Syahrul et al., 2021). According to the most recent Global Burden of Disease study, stroke is the second leading cause of death worldwide and a primary cause of long-term disability in adults (GBD 2019 Stroke Collaborators, 2021). With the accelerating pace of global population aging, the disease burden of stroke is projected to intensify worldwide. Projections indicate that by 2050, global stroke-related mortality will increase by 50% (Feigin et al., 2025), imposing a substantial economic and healthcare burden on families and society. Consequently, in-depth exploration of stroke pathophysiological mechanisms and the identification of novel targets for prevention, treatment, and rehabilitation have emerged as critical priorities in contemporary research.

The gut microbiota constitutes a complex microbial ecosystem residing in the human gastrointestinal tract, and the collective sum of its genetic material—the microbiome—is often referred to as the “second human genome”. These microorganisms are not only involved in fundamental physiological processes—including digestion, nutrient absorption, and vitamin synthesis—but also function collectively as a crucial metabolic “organ” within the human body. Through the production of bioactive metabolites—including short-chain fatty acids (SCFAs), secondary bile acids, and neurotransmitter precursors—they play a pivotal role in regulating host nutritional metabolism, inflammatory responses, and immune system function (Krautkramer et al., 2021; Bemark et al., 2024; Cantón et al., 2024). The diverse array of microorganisms within this ecosystem exist in a state of dynamic interdependence and competition, maintaining a compositional balance that is critical for gut homeostasis.

In recent years, advances in the conceptual framework of the “gut-brain axis” have increasingly revealed the pivotal role of the gut microbiota in the pathophysiology of stroke. The gut-brain axis is a bidirectional communication pathway that integrates signals between the central nervous system and the gastrointestinal tract through complex and dynamic interactions (Osadchiy et al., 2019). A growing body of evidence indicates that stroke not only induces gut microbiota dysbiosis and compromises intestinal barrier integrity, but that this dysbiosis, in turn, exacerbates brain injury through a self-perpetuating cycle involving multiple mechanisms. These include the modulation of immune and inflammatory responses, alterations in the production of microbial metabolites (e.g., short-chain fatty acids, SCFAs), and disruption of neurotransmitter homeostasis. Consequently, these effects have a significant impact on patient prognosis (Loh et al., 2024). Thus, the gut microbiota represents a critical modifiable factor influencing both the pathogenesis and progression of stroke.

As research into the relationship between stroke and the gut microbiota has intensified, the volume of relevant publications has increased dramatically. However, navigating this expansive and rapidly growing body of literature presents a significant challenge, often requiring considerable time for researchers to assimilate the latest advances in the field. While observational studies and systematic reviews have synthesized key findings from specific perspectives, they are inherently limited in their capacity to comprehensively delineate the field’s overarching knowledge structure, developmental trajectory, and evolving research trends (Li et al., 2024a). Furthermore, traditional narrative reviews are generally unable to provide a comprehensive, quantitative overview of key bibliometric indicators, such as the distribution of contributions by country/region, leading institutions, research cluster formation, and patterns of scientific collaboration. Consequently, to address the inherent limitations of traditional reviews, bibliometric methods have gained widespread recognition and application within the biomedical research community as a valuable complementary approach (Montazeri et al., 2023).

Stroke is broadly classified into two major subtypes—ischemic and hemorrhagic—which differ substantially in their etiology, pathophysiological mechanisms, and clinical outcomes. Ischemic stroke accounts for approximately 85% of all cases and is primarily associated with atherosclerosis and thrombus formation (Bogenschutz et al., 2025). The primary pathological mechanism involves the rupture of vulnerable atherosclerotic plaques, which exposes prothrombotic sub-endothelial components to the circulating blood. This event activates platelets and initiates the coagulation cascade, ultimately culminating in thrombus formation and vascular occlusion (Kosyakovsky et al., 2025). In contrast, the pathological progression of hemorrhagic stroke is typically divided into two distinct stages: primary and secondary injury. Primary injury results from the rupture of cerebral vessels, leading to blood extravasation into the brain parenchyma or subarachnoid space. This forms a hematoma, increases intracranial pressure, and causes immediate mechanical damage to neural tissue. Secondary injury encompasses a series of complex cascade reactions triggered by cytotoxic blood components within the hematoma, notably iron released from lysed erythrocytes. These deleterious processes primarily include oxidative stress, neuroinflammation, disruption of the blood–brain barrier, and the development of cerebral edema. The combined effects of these mechanisms exacerbate perihematomal tissue damage and contribute to sustained neurological deficits (Shao et al., 2019; Sun et al., 2021). Current evidence indicates that the role of the gut microbiota in the pathogenesis of ischemic stroke has been relatively well characterized, whereas its involvement in hemorrhagic stroke remains an emerging area of investigation. Therefore, building upon a systematic synthesis of the current research landscape, future investigations should aim to delineate the subtype-specific characteristics of gut microbiota involvement. Such granularity is essential for providing more precise, subtype-tailored guidance for clinical practice.

Bibliometrics is a research methodology that employs statistical, mathematical, and information science approaches to conduct both qualitative and quantitative analyses of the literature within a specific field. This methodology can effectively identify core research teams and potential collaborators, delineate research hotspots, and provide a macroscopic overview of disciplinary trends, thereby furnishing critical evidence for charting important future research directions (Thompson and Walker, 2015). In recent years, the exponential growth of biomedical literature, coupled with the rapid development of accessible bibliometric software tools like CiteSpace and VOSviewer, has propelled bibliometrics to the forefront of research evaluation in the biomedical field (van Eck and Waltman, 2017; Cheng et al., 2024). Using the gut microbiota as an illustrative example, previous bibliometric studies have spanned a diverse range of biomedical fields, including oncology (Long et al., 2023; Wu et al., 2023), autoimmune diseases (Ouyang et al., 2024; Zhao et al., 2024), metabolic diseases (Zhang et al., 2022), and musculoskeletal disorders (He et al., 2025). Within the field of neurology, bibliometric analyses have mapped the research landscape and identified hotspots in conditions such as Alzheimer’s disease (Li et al., 2022), Parkinson’s disease (Li et al., 2024b), and depression (Xu et al., 2025). However, to date, there has been a notable absence of systematic bibliometric analyses mapping the research hotspots and developmental trends at the intersection of gut microbiota and stroke. To address this gap, the present study employs multiple bibliometric tools to systematically analyze the relevant literature. The primary objectives are: (1) to delineate the overall publication trends from January 1, 2000, to August 31, 2025; (2) to identify the principal contributors at the country, institutional, and funding agency levels; and (3) to elucidate the developmental trajectories and evolutionary trends within this research domain.

## 2 Materials and methods

The methodology of this study followed the Cochrane manual, and the report outlined in this study complied with the PRISMA2020 checklist.

### 2.1 Data acquisition and search strategy

**Figure 1:**
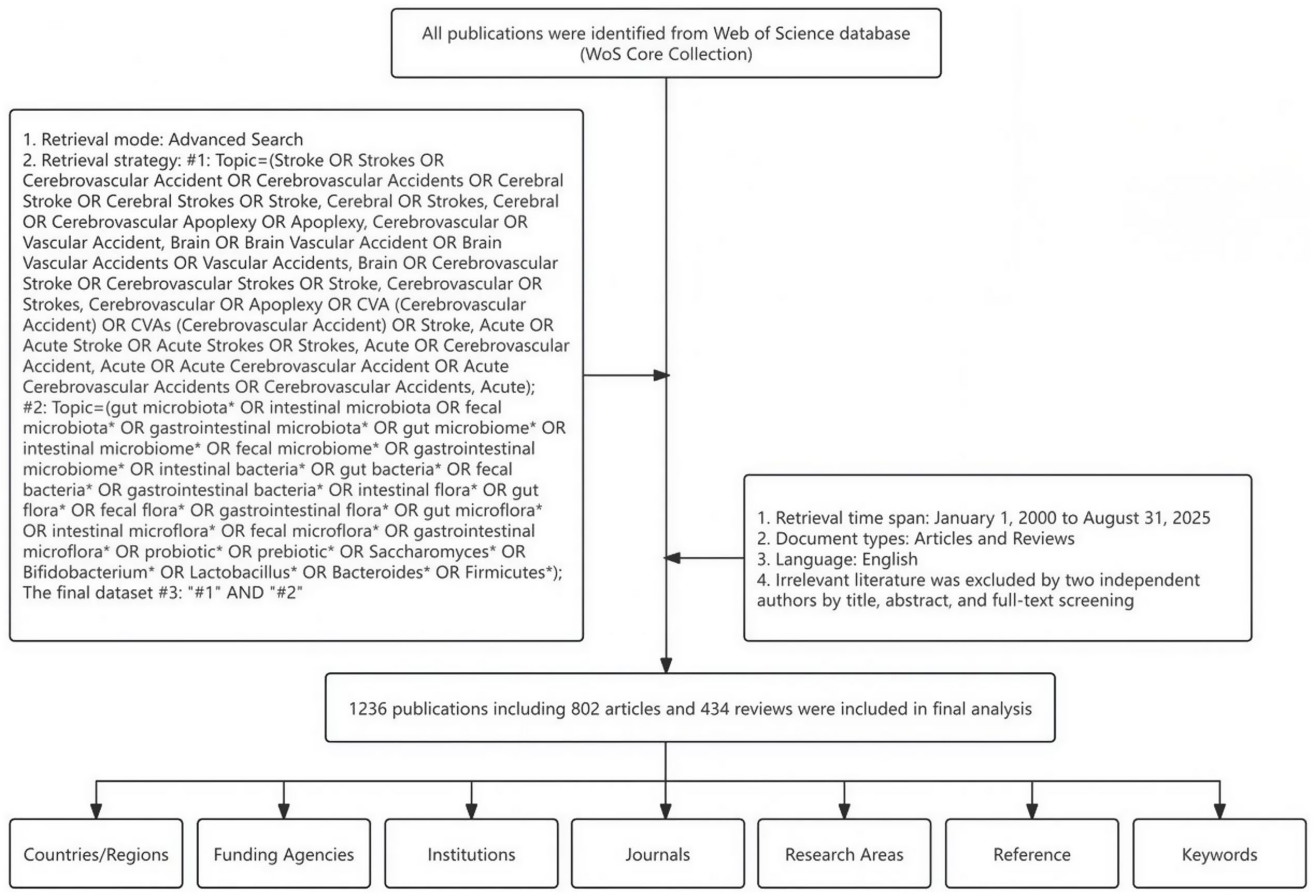
Literature screening process.

The Web of Science Core Collection (WOSCC), provided by Clarivate Analytics, is an authoritative citation database covering multiple disciplines, including natural sciences, social sciences, arts, and humanities. It is widely recognized as one of the most reliable and publisher-independent citation databases in the world (Wang et al., 2025). It is currently widely used in bibliometric research and scientific writing. The data retrieval time range is limited to January 1, 2000-August 31, 2025. Using the advanced search mode, focusing on the keywords “stroke” and “gut microbiota,” the specific search formula is as follows: TS = (Stroke OR Strokes OR Cerebrovascular Accident OR Cerebrovascular Accidents OR Cerebral Stroke OR Cerebral Strokes OR Stroke, Cerebral OR Strokes, Cerebral OR Cerebrovascular Apoplexy OR Apoplexy, Cerebrovascular OR Vascular Accident, Brain OR Brain Vascular Accident OR Brain Vascular Accidents OR Vascular Accidents, Brain OR Cerebrovascular Stroke OR Cerebrovascular Strokes OR Stroke, Cerebrovascular OR Strokes, Cerebrovascular OR Apoplexy OR CVA (Cerebrovascular Accident) OR CVAs (Cerebrovascular Accident) OR Stroke, Acute OR Acute Stroke OR Acute Strokes OR Strokes, Acute OR Cerebrovascular Accident, Acute OR Acute Cerebrovascular Accident OR Acute Cerebrovascular Accidents OR Cerebrovascular Accidents, Acute) AND TS = (gut microbiota* OR intestinal microbiota OR fecal microbiota* OR gastrointestinal microbiota* OR gut microbiome* OR intestinal microbiome* OR fecal microbiome* OR gastrointestinal microbiome* OR intestinal bacteria* OR gut bacteria* OR fecal bacteria* OR gastrointestinal bacteria* OR intestinal flora* OR gut flora* OR fecal flora* OR gastrointestinal flora* OR gut microflora* OR intestinal microflora* OR fecal microflora* OR gastrointestinal microflora* OR probiotic* OR prebiotic* OR Saccharomyces* OR Bifidobacterium* OR Lactobacillus* OR Bacteroides* OR Firmicutes*). The document types were limited to “articles” and “reviews,” and the search results were restricted to English-language documents. To ensure consistency and accuracy in data extraction and download, and to minimize differences caused by data updates, all searches were completed on the same day. Two researchers independently screened the documents, reviewing the titles, abstracts, and full texts. The exclusion criteria were as follows: (1) Duplicate documents; (2) Documents mistakenly labeled by WOSCC as non-articles or non-reviews; (3) Studies unrelated to stroke and gut microbiota. Any disagreements during the screening process were resolved by a third researcher. The literature screening process is shown in Figure 1.

### 2.2 Data extraction

Based on the above search strategy, the selected documents were downloaded in the “Full Record and Cited References” format and exported in text format. Microsoft Office Excel 2021 was used to statistically analyze bibliometric indicators such as the number of publications per year, citation frequency, distribution by country/region, institutional information, author information, funding agencies, journal information, keywords, and references. The total number of citations, average number of citations, and H-index were evaluated using the citation report in WOSCC. The journal impact factor (IF) and quartile (Q1-Q4) data were obtained from the 2025 Journal Citation Reports (JCR)^1^ The H-index, proposed by Hirsch, is a major indicator for measuring the impact of researchers’ publications in the scientific field (Hirsch, 2005). It is defined as the number n of papers published by a scholar, each of which has been cited at least n times. Within the same discipline, the JCR categorizes journals into four quartiles based on their impact factors: the top 25% of journals are classified as Q1, the next 25–50% as Q2, and so on. To address the issue of fragmented regional classification in the WOSCC database, this study integrates publications from multiple regions into national-level data (For example, England, Northern Ireland, Scotland, and Wales are combined as the United Kingdom; Chinese Mainland and Taiwan are combined as China).

### 2.3 Data analysis

Microsoft Office Excel 2021 and the R language (v 4.1.0) were used for descriptive data analysis, chart drawing, and curve fitting. Excel was employed to visualize the trends in annual publication and citation volumes, and exponential, linear, logarithmic, and polynomial curve fitting methods were applied. The optimal model was selected by comparing the coefficient of determination (R^2^). The formula for calculating the average annual growth rate of publications is as follows: Annual growth rate = [(Number of publications in 2025 ÷ Number of publications in 2000)^(1/25) − 1] × 100 (Wu et al., 2022). Bibliometric analysis and visualization were conducted using Citespace (v6.4. R1) and VOSviewer (v1.6.20). Citespace is an open-source bibliometric analysis software based on Java, with the following specific parameter settings: (1) Time slicing from 2000 to 2025, with each year as a time slice; (2) Node types include keywords, authors, institutions, and references; (3) Network pruning is performed using the Pathfinder method; (4) Top Nperslice: for author node type, “Top Nperslice = 30”; for reference node type, “Top Nperslice = 50”. VOSviewer, a software tool equipped with text mining capabilities, can extract key terms and relational data from large volumes of literature, generating network, overlay, and density visualizations to map the scholarly landscape. In this study, VOSviewer was employed to analyze countries/regions, institutions, journals, and keywords. For each unit of analysis, minimum occurrence thresholds were established to ensure the inclusion of sufficiently frequent items. To enhance accuracy and avoid redundancy, synonyms and variant forms of keywords (e.g., abbreviations and plural forms) were consolidated using a customized thesaurus file prior to analysis.

When conducting country/region-based publication counts, we employed the whole counting method. Under this approach, a single paper co-authored by researchers from multiple countries or regions is counted toward the total publication output of each participating country or region. Consequently, the sum of publications attributed to individual countries or regions exceeds the actual total number of documents (*N* = 1,236). This methodology provides a comprehensive reflection of the level of participation and contribution of each country or region within the research field under investigation.

### 2.4 Multi-database validation

To assess the robustness and comprehensiveness of the findings derived from the WOSCC database, supplementary literature searches were performed in the Scopus and PubMed databases. The search strategy, including keywords, Boolean operators, and time frame, was designed to be consistent with that used for WOSCC while being adapted to the specific syntax requirements of each database. The same inclusion and exclusion criteria applied to the WOSCC dataset were employed for the Scopus and PubMed searches to ensure the comparability of results across databases.

Recognizing the inherent differences in data structure, indexing policies, and functional capabilities among the three databases, a hierarchical and adaptive strategy was implemented for this cross-database validation. First, a basic consistency verification was conducted to compare the three databases. For this purpose, annual publication trends, the ranking of major countries/regions by output, and high-frequency keywords were extracted and compared across all three databases. The Pearson correlation coefficient was calculated for the annual publication counts to quantitatively assess the consistency of temporal trends between database pairs. Second, characteristic differences between the databases were analyzed. This analysis focused on two aspects: (1) terminological preferences, inferred from database-specific high-frequency keywords; and (2) journal coverage characteristics, compared to elucidate the complementary nature of the academic resources indexed by each database.

## 3 Results

### 3.1 Global perspective

After systematic literature search, screening, and data cleaning, a total of 1,236 documents were finally included. Analysis using the R language revealed that research on stroke and gut microbiota involved 6,756 researchers from 1,853 institutions across 150 countries or regions, and covered 546 different journals. The total number of citations for these documents reached 56,363, with an average of 45.56 citations per document. The overall H-index of the included documents during the study period (January 1, 2000–August 31, 2025) was 104, reflecting excellent and enduring academic influence. The document screening process is shown in Figure 1.

### 3.2 Analysis of disciplinary categories

**Figure 2:**
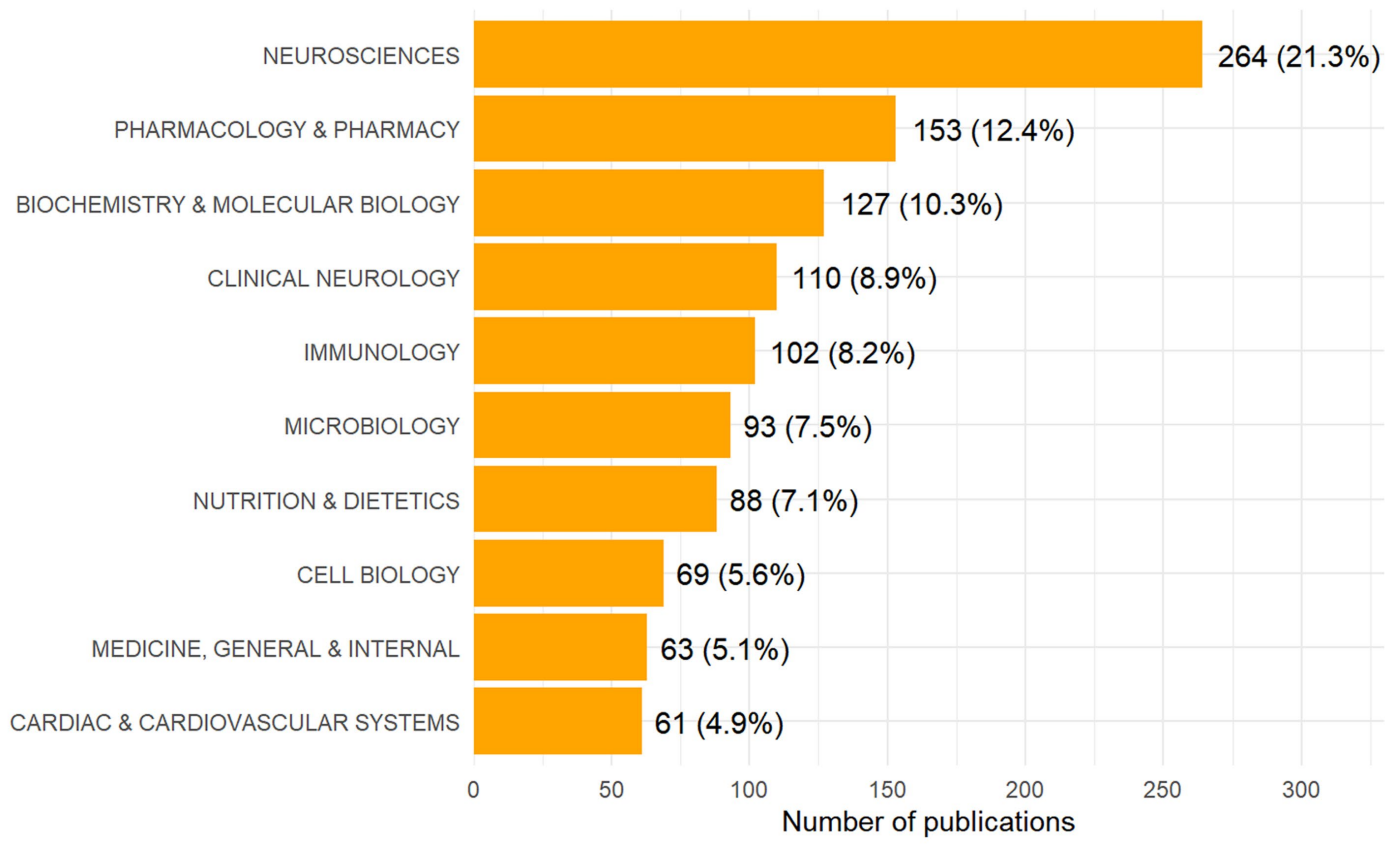
Disciplines ranked by publication output.

According to the WOS subject classification, the 1,236 documents on stroke and gut microbiota research cover 89 different subject categories. To clarify the distribution of these categories, the top 10 subjects ranked by the number of publications and their respective proportions are shown in Figure 2. Among them, “Neurosciences” has the highest number of publications, with a total of 264 articles (21.3%); followed by “Pharmacology & Pharmacy” and “Biochemistry & Molecular Biology”, with 153 (12.4%) and 127 (10.3%) publications, respectively. In addition, “Clinical Neurology” and “Immunology” also demonstrate high academic output.

Notably, Figure 2 illustrates the inherently interdisciplinary nature of research at the intersection of stroke and the gut microbiota. The most frequently represented disciplines extend beyond neurology to encompass pharmacology, biochemistry, immunology, microbiology, nutrition, cell biology, and cardiovascular medicine, reflecting a strong trend toward interdisciplinary integration and collaboration. This synergistic approach not only advances a holistic understanding of the stroke-gut microbiota interaction but also establishes a comprehensive research continuum spanning from fundamental molecular mechanisms to translational clinical applications. Lines of evidence from diverse disciplines mutually corroborate and complement one another, thereby transforming the once-vague “gut-brain axis” hypothesis into a robust scientific framework characterized by well-defined molecular foundations, cellular targets, and potential intervention strategies.

### 3.3 Analysis of trends in publication outputs

**Figure 3:**
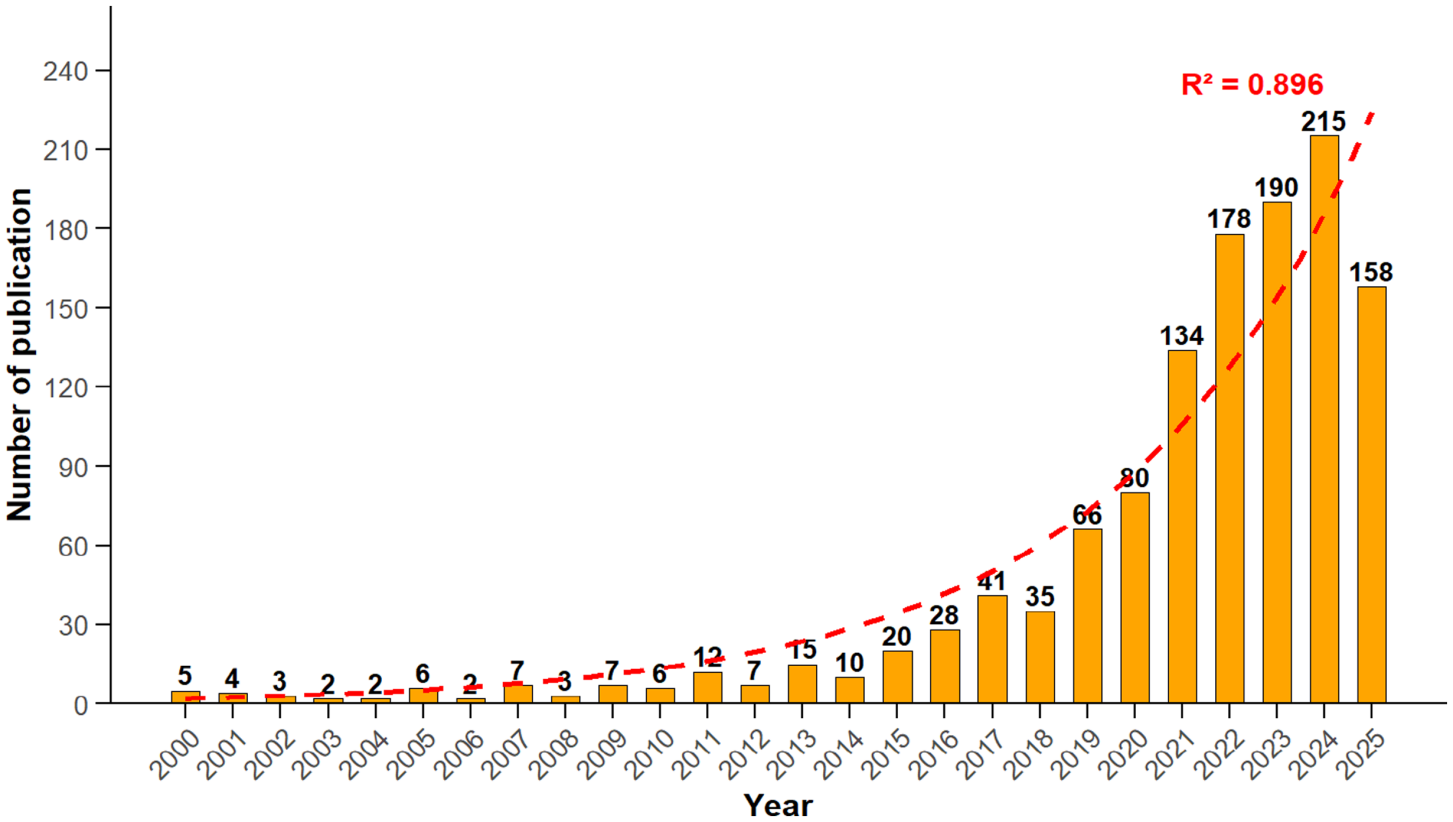
Annual publication trends in the field of stroke and gut microbiota research.

The number of publications in different periods directly reflects the development trend and evolution of the field (Wang et al., 2024). Among the 1,236 included documents (including 802 research papers and 434 reviews), the annual publication situation of literature related to gut microbiota and stroke from January 1, 2000, to August 31, 2025, is shown in Figure 3. Obviously, research in this field shows a significant growth trend, especially in the past 5 years, with an average annual publication volume of more than one hundred papers, reaching a peak in 2024 (data for 2025 is up to August 31, 2025). R^2^ is a key statistical indicator measuring the goodness of fit of the trend line to the actual data. In this study, R^2^ = 0.896, indicating that the fitted trend line can reliably reflect the real change pattern of publication output. The annual growth rate of publications is 20.6%.

While research on the relationship between stroke and the gut microbiota has experienced exponential growth over the past decade, it is important to acknowledge that pioneering studies from the early stages laid the essential groundwork for this expansion. The rapid development of this field can be largely attributed to advances in high-throughput sequencing technologies and the concurrent refinement of the “gut-brain axis” theoretical framework. Given the substantial global burden of stroke and the promising therapeutic potential of microbiota-based interventions, it is anticipated that both the annual volume of publications and the level of researcher engagement in this domain will continue to grow steadily in the coming years.

### 3.4 Analysis of countries/regions

**Figure 4:**
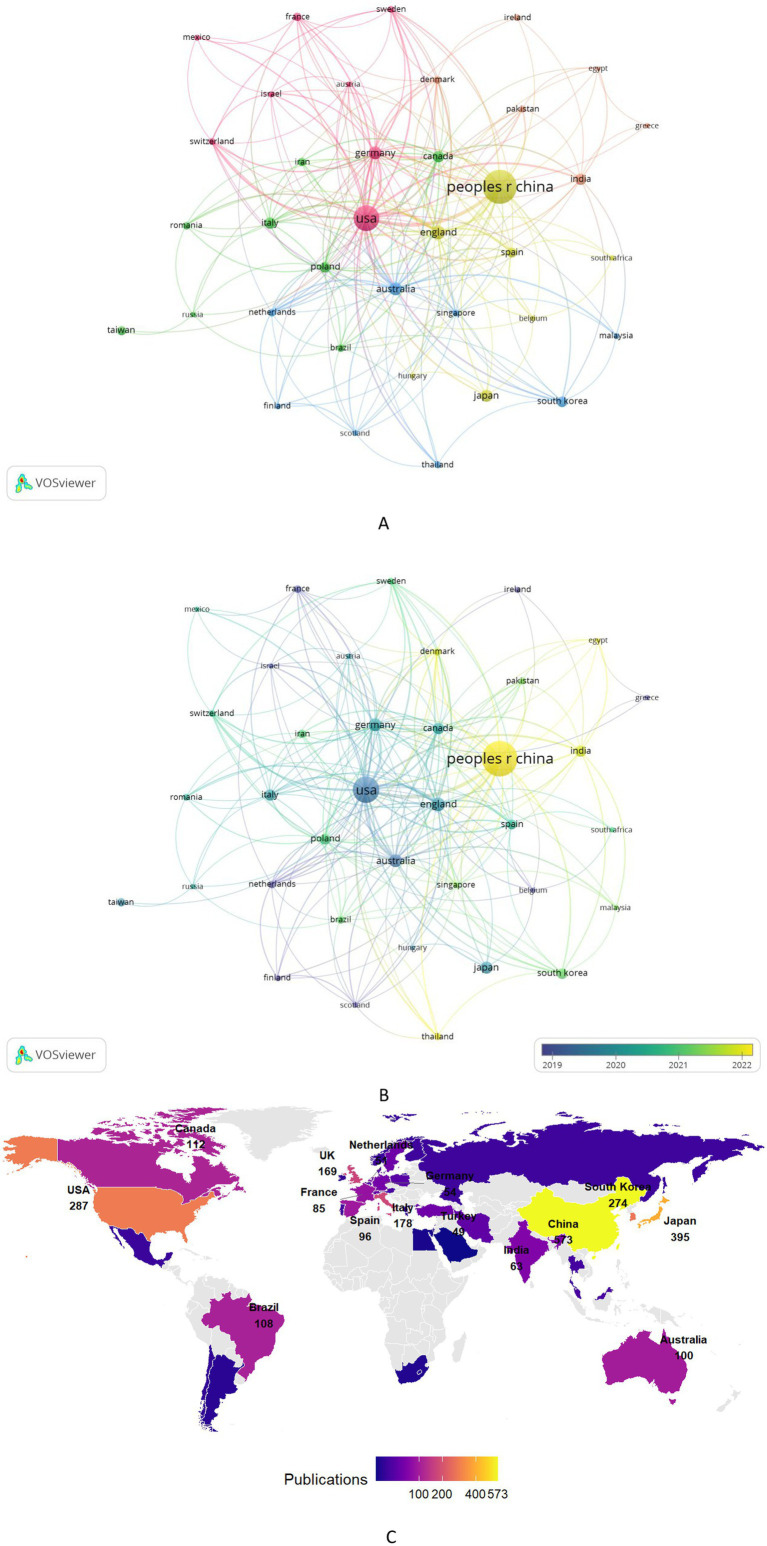
Analysis collaboration network. **(A)** Visualization of the countries/regions collaboration network. Nodes (circles) represent individual countries/regions, with node size proportional to their publication volume. Edges (lines) denote collaborative relationships between countries/regions, where edge thickness reflects the strength of collaboration. **(B)** Time-overlay visualization of the countries/regions collaboration network. Node color intensity corresponds to the average publication year (warmer hues indicate more recent contributions). **(C)** Geographical distribution map of different countries/regions.

Figure 4A is a map of the global research collaboration on stroke and gut microbiota generated using VOSviewer. Publications in this field cover 75 countries/regions. As shown in Table 1, among the top 10 countries/regions in terms of publication volume, China leads by a large margin with 573 publications, far ahead of Japan (395 publications), the United States (287 publications), South Korea (274 publications), and Italy (178 publications). China also ranks high in terms of citation counts, indicating that it has invested substantial scientific resources in this field and is a core driving force for its development. However, its average citation per paper is relatively low, suggesting that its influence may stem from a large number of publications. It is worth noting that although the United Kingdom and Italy have lower publication volumes than China, their total link strengths are 238 and 216, respectively, both higher than China’s (108). In particular, the United Kingdom has the highest total link strength, showing that it plays an important hub role in the global collaboration network and is likely to produce high-quality research outcomes. In addition, Asian countries are active in this research field, with China, Japan, and South Korea ranking in the top four, reflecting that this research topic has received high attention and wide coverage in the Asian region.

**Table 1:** Top 10 countries or regions ranked by publication output.

Rank	Country/Region	Publications	Total citations	Total link strength
1	China	573	11,521	108
2	Japan	395	24,918	203
3	USA	287	9,242	70
4	South Korea	274	8,776	67
5	Italy	178	13,238	216
6	United Kingdom	169	10,183	238
7	Canada	112	6,652	100
8	Brazil	108	2,657	69
9	Australia	100	4,290	119
10	Spain	96	3,166	123

As shown in Figure 4B, the temporal overlay visualization analysis of the national collaboration network reflects the average publication years of research articles from high-output countries/regions. The United States, as a traditional leading force, has an earlier average publication year for its research outcomes and is at the center of the global collaboration network, maintaining extensive and close cooperation with various countries. In contrast, China, as an emerging core, indicates that its output is mainly concentrated in recent years. European countries (such as the United Kingdom, Germany, and Italy) have formed a mature collaboration network with close internal connections. Overall, the global research power has evolved from a single-center model centered on the United States to a multipolar collaborative model with North America, Europe, and East Asia standing side by side, reflecting the globalization and diversification trend of this research field.

Figure 4C displays the geographical distribution of research output on stroke and gut microbiota, showing regional clustering of publications in North America, East Asia, and Europe. In North America, the United States (with 287 publications) and Canada (with 112 publications) form an active research area. China, with 573 publications, stands out as the global core, while South Korea (with 274 publications) also plays a significant role, highlighting the high level of participation and influence of East Asia in this research field. European countries exhibit strong intra-regional collaborative features, with countries like the United Kingdom (with 169 publications) not only maintaining close regional cooperation but also engaging in extensive international collaboration with major research powers such as the United States and China. This multi-level collaborative network not only expands the scope of academic partnerships but also effectively promotes cross-cultural knowledge exchange and innovation.

### 3.5 Analysis of institutions and authors

It was found that a total of 6,756 individuals from 1,853 institutions have participated in research on stroke and gut microbiota. To identify the core entities with high output and high impact, Tables 2, 3 list the top 10 institutions and authors in terms of publication volume, respectively. The statistical analysis shows that Southern Medical University (China) ranks first globally with 47 papers. In addition, eight of the top 10 institutions are from China, namely Zhejiang University (26 publications), Capital Medical University (24 publications), Central South University (22 publications), Wenzhou Medical University (22 publications), Nanjing Medical University (20 publications), University of Chinese Academy of Sciences (18 publications), and Harbin Medical University (17 publications). This distribution pattern demonstrates China’s dominant position in the research field of stroke and gut microbiota, which is consistent with its leading advantage in overall national publication volume. From the perspective of authors, Wang Y ranks first with 20 publications, highlighting his core contribution and academic activity in this field. Yin J and Li J are tied for second place with 20 publications each. It is worth noting that although Hazen S. L. has published only 19 papers, his total citation count is as high as 9,711, indicating that his research findings have significant academic influence. Li L, with an extremely high citation count of 6,312, further confirms the core value and academic status of his research work.

**Table 2:** Top 10 institutions ranked by publication output.

Rank	Institution	Country	Publications	Percentage
1	Southern Medical University	China	47	3.80%
2	Zhejiang University	China	26	2.10%
3	Capital Medical University	China	24	1.94%
4	Central South University	China	22	1.78%
5	Wenzhou Medical University	China	22	1.78%
6	Cleveland Clinic	USA	21	1.70%
7	Nanjing Medical University	China	20	1.62%
8	University of Chinese Academy of Sciences	China	18	1.46%
9	Harbin Medical University	China	17	1.37%
10	Harvard Medical School	USA	17	1.37%

**Table 3:** Top 10 authors ranked by publication output.

Rank	Author	Publications	Percentage	Total citations
1	Wang Y	22	1.78%	480
2	Yin J	20	1.62%	1,525
3	Li J	20	1.62%	412
4	Hazen S. L.	19	1.54%	9,711
5	Liu JM	18	1.46%	442
6	Li L	18	1.46%	6,312
7	He Y	16	1.29%	1,512
8	Zhang Y	16	1.29%	249
9	Zhou HW	15	1.21%	1,545
10	Sun J	15	1.21%	456

### 3.6 Analysis of journals

**Figure 5:**
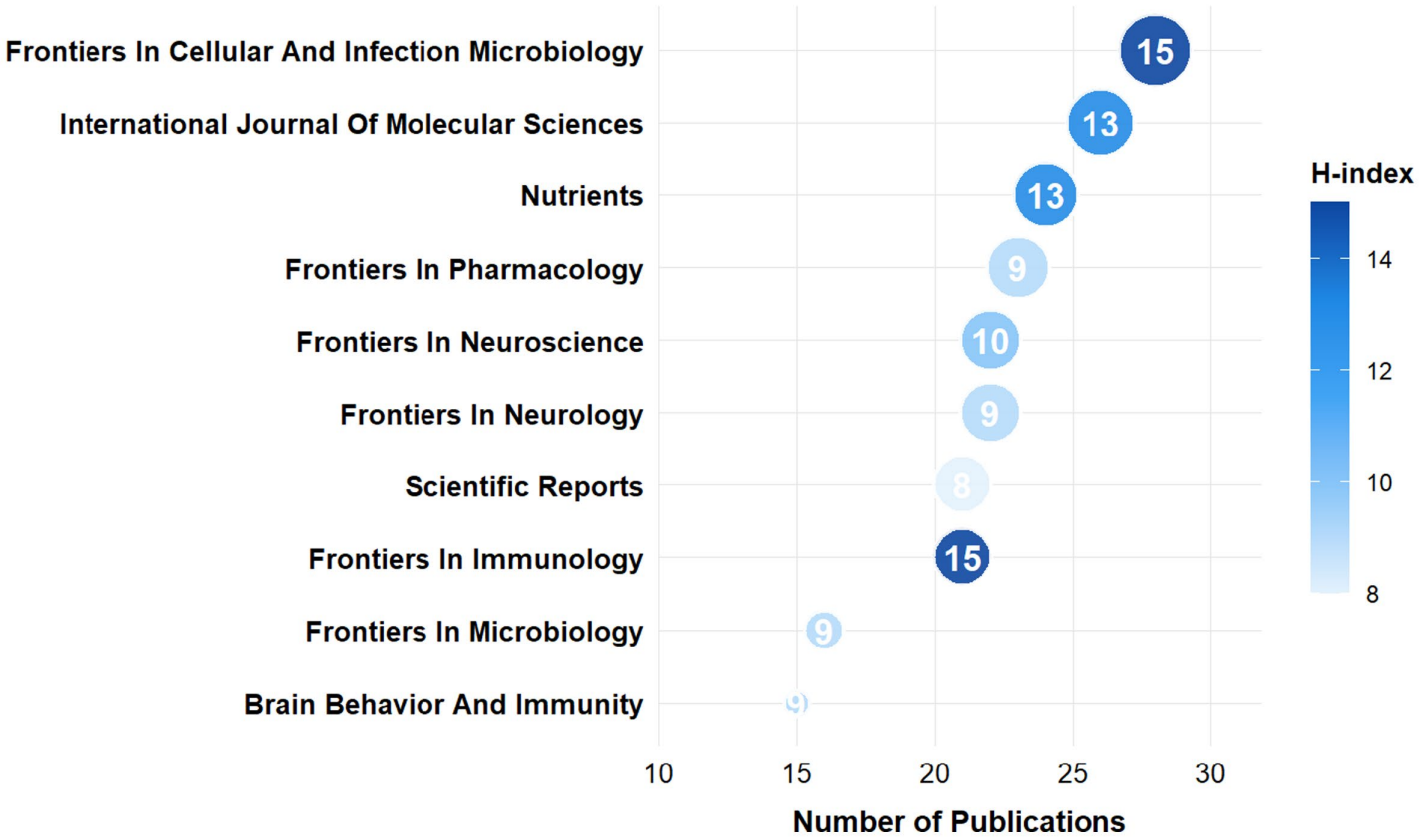
The top 10 journals in terms of publication output.

Figure 5 shows the top 10 journals in terms of publication volume on stroke and gut microbiota. Among these journals, the one with the most related articles is “Frontiers in Cellular And Infection Microbiology”, followed by “International Journal Of Molecular Sciences” and “Nutrients.” Most of these journals are classified as Q1 or Q2, with “Brain Behavior And Immunity” having the highest impact factor (IF) of 7.6. In terms of H-index and ACI, “Frontiers in Cellular And Infection Microbiology” has the highest H-index of 15, while “Brain Behavior And Immunity” has the highest ACI of 37.27.

**Figure 6:**
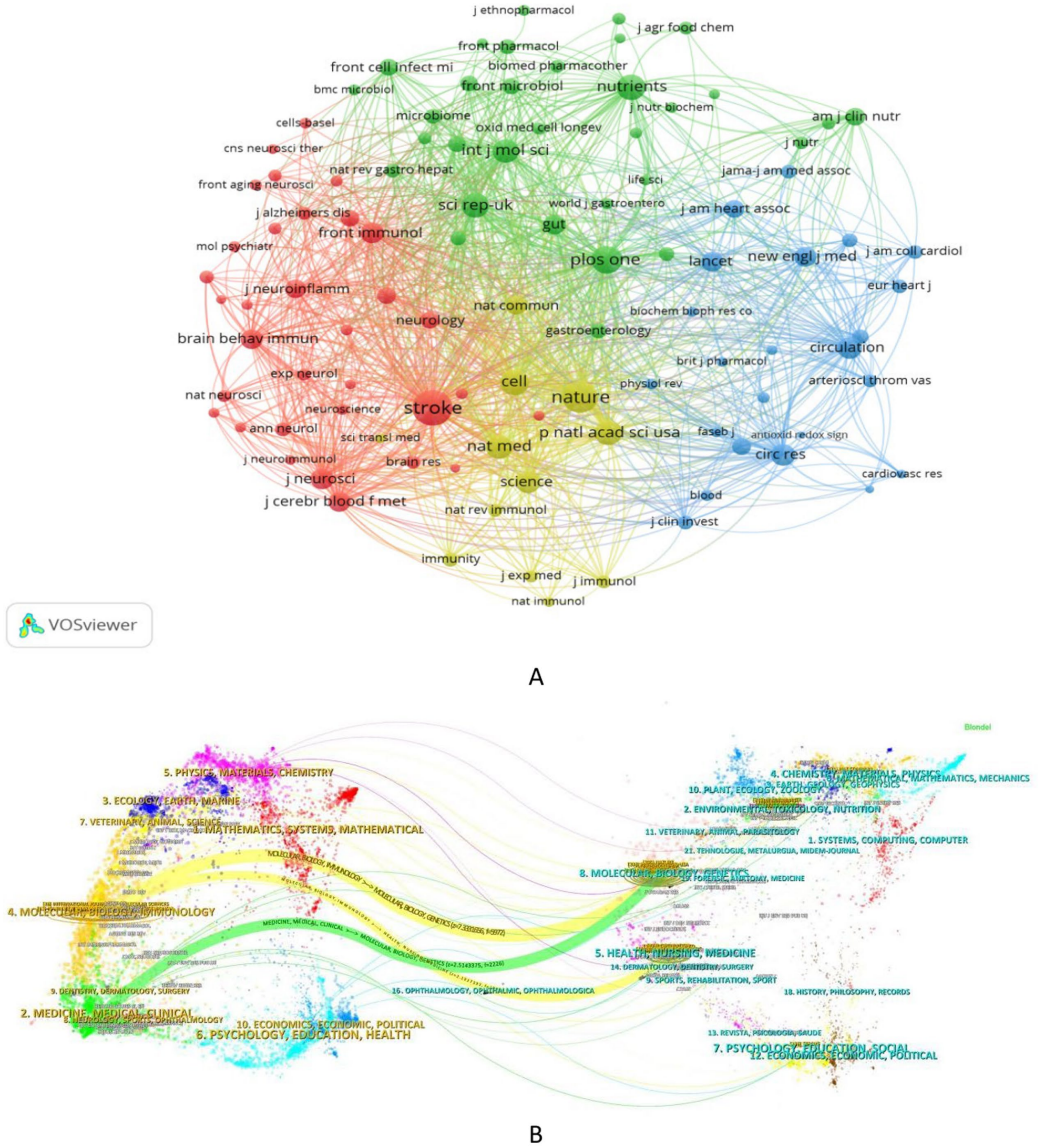
Analysis of co-cited journals. **(A)** Visualization of journal co-citation network. Nodes represent journals, with their size proportional to co-citation frequency. The links between nodes represent co-citation relationships. Node color denotes different clusters, and its centrality reflects the journal’s role as a bridge within the network, with higher values indicating greater importance in connecting disparate research fields. **(B)** The dual-map overlay of journals. The left side represents citing journals, while the right side represents cited journals. The curved lines between them represent citation links, indicating the citation paths, with the thickness of the lines proportional to citation frequency.

Journal co-citation refers to the simultaneous citation of two or more journals by one or more documents. The higher the frequency, the closer the association between the journals (van Eck and Waltman, 2010), which is a key indicator for assessing academic reputation and disciplinary influence. Figure 6A and Table 4 show the journal co-citation network and the top 10 journals in terms of co-citation frequency in this field. “Stroke” ranks highest with 2,424 co-citations, followed by “Nature” (1,892 co-citations) and “PloS One” (1,488 co-citations). It is worth noting that most of these journals are top-tier journals with extremely high impact factors (e.g., “Nature Medicine” IF: 50.0, “Nature” IF: 48.5) and are mostly classified in the JCR Q1 quartile, indicating the high quality and authority of research in this field.

**Table 4:** Top 10 co-cited journals in terms of number of co-citations.

Rank	Co-cited journal	Co-citation frequency	Centrality	IF(2024)	JCR quartile (2024)
1	Stroke	2,424	0.278	8.9	Q1
2	Nature	1892	0.167	48.5	Q1
3	PloS One	1,488	0.000	2.6	Q2
4	Cell	1,256	0.083	42.5	Q1
5	Nutrients	1,239	0.167	5.0	Q1
6	Scientific Reports	1,230	0.000	3.9	Q1
7	Proceedings of the National Academy of Sciences of the United States of America	1,187	0.000	9.1	Q1
8	Nature Medicine	1,148	0.167	50.0	Q1
9	International Journal of Molecular Sciences	1,077	0.000	4.9	Q1
10	Circulation	924	0.083	38.6	Q1

In addition, co-citation patterns reflect interdisciplinary collaboration. Journals in the fields of stroke, neuroscience, basic medicine, and nutrition dominate the rankings, which is highly consistent with the interdisciplinary nature of stroke and gut microbiota research. Centrality analysis shows that “Stroke” (centrality = 0.278) is the “core bridge” connecting different disciplinary fields. In contrast, journals such as “PloS One” and “Scientific Reports” have a centrality of 0, indicating that despite their high co-citation frequencies, their bridging role in the network is relatively weak and their disciplinary connection scope is relatively limited.

The dual-map overlay is a visualization tool that illustrates the citation relationships between citing and cited journals (Chen and Leydesdorff, 2014). This analytic tool provides a panoramic view of how research within a specific domain is interconnected with other disciplines, clearly revealing the intersections and flows of knowledge across the broader scientific landscape. Figure 6B presents the dual-map overlay for the field of stroke and gut microbiota research, as generated by CiteSpace. In this visualization, journal citation links are filtered using the z-score indicator to emphasize the most significant pathways of interdisciplinary knowledge flow. The primary disciplines represented among the cited journals include molecular biology, genetics, health, nursing, general medicine, as well as neurology and clinical neurology. Three major citation trajectories emanate from these foundational disciplines and converge upon cutting-edge research areas that are deeply integrated with molecular biology, immunology, general medicine, neurology, and clinical disciplines. Furthermore, methodological disciplines—including chemistry, physics, and mathematics—along with fields related to prognosis and rehabilitation, such as psychology, education, and health sciences, are poised to emerge as key interdisciplinary intersections and promising new frontiers that will drive the future evolution of this research domain.

### 3.7 Analysis of co-cited references

**Figure 7:**
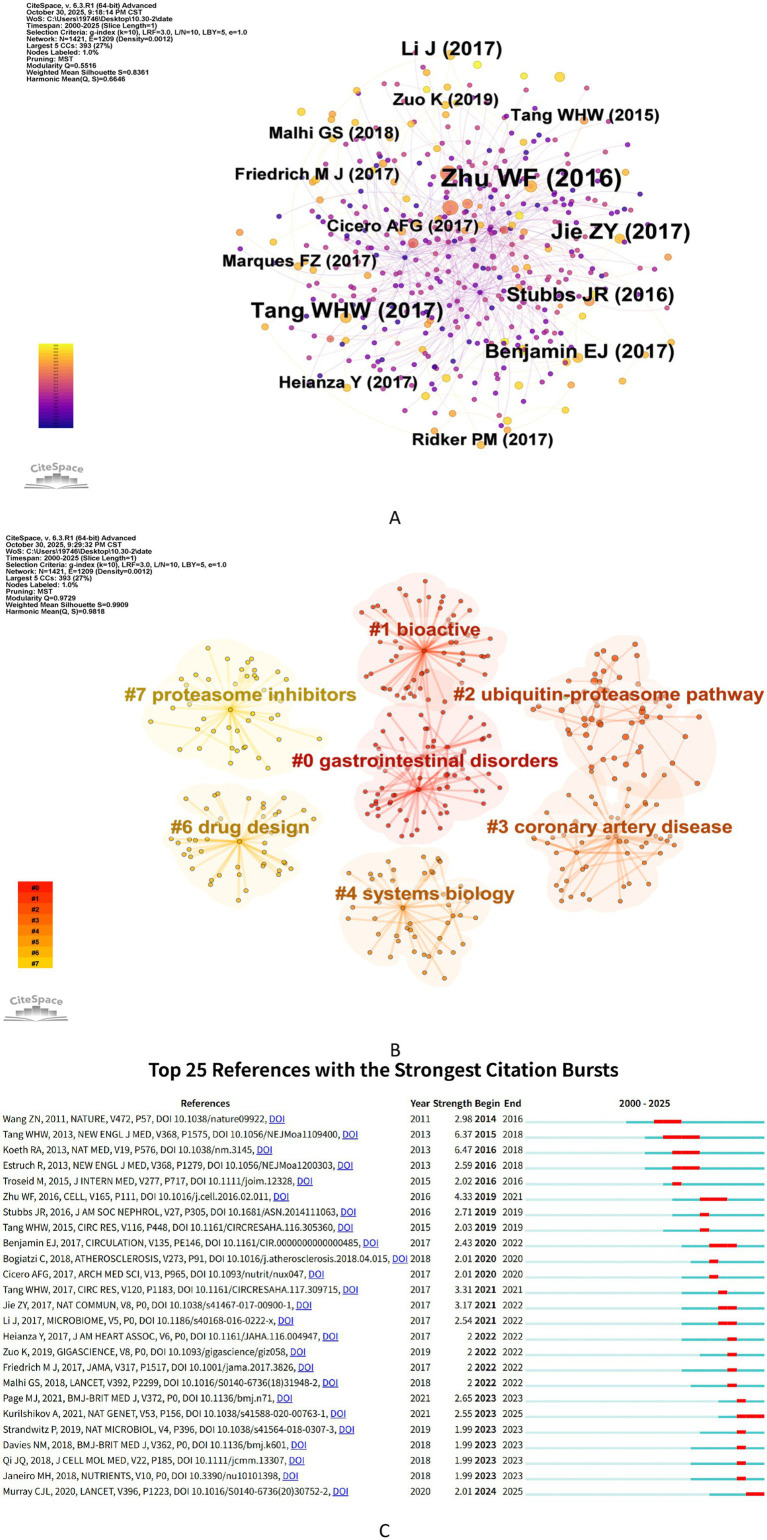
Analysis of co-cited reference. **(A)** Co-citation network visualization of references. Nodes represent cited references, with their size proportional to co-citation frequency. Links between nodes denote co-citation relationships, and their thickness indicates the strength of association. The color of nodes represents distinct thematic clusters derived from the co-citation patterns. **(B)** Clustering network analysis of references. **(C)** Top 25 references with the strongest citation bursts. “Strength” quantifies the intensity of citation bursts, while “Begin” and “End” demarcate the burst duration (indicated by red segments). Longer red spans correspond to sustained periods of heightened citation activity.

Co-citation analysis identifies core literature and research hotspots by statistically counting the frequency with which two documents are cited together. It can effectively reveal the knowledge structure and development trends of a discipline (Feng et al., 2024). Figure 7A shows the co-citation network, and Table 5 summarizes the top 10 co-cited documents, all published between 2011 and 2020, with co-citation frequencies exceeding 100 times. The most frequently co-cited document is the article published in 2016 by Singh et al. (2016) in the “Journal of Neuroscience”. This study revealed that post-stroke gut microbiota dysbiosis exacerbates neuroinflammation and worsens brain injury outcomes by promoting the polarization and migration of pro-inflammatory T cells to the brain, confirming the key role of the “brain-gut-immune axis” in stroke pathogenesis. It also proposed that fecal microbiota transplantation can reverse dysbiosis, modulate immune responses, and improve outcomes, providing a new therapeutic target for stroke treatment.

**Table 5:** The top 10 highly cited papers on the co-citation references.

Rank	Title	First author	Journal	Year	Co-citation frequency	Citation density
1	Microbiota Dysbiosis Controls the Neuroinflammatory Response after Stroke	Singh, V.	Journal of Neuroscience	2016	294	32.67
2	Commensal microbiota affects ischemic stroke outcome by regulating intestinal γδ T cells	Benakis, C	Nature Medicine	2016	254	28.22
3	Dysbiosis of Gut Microbiota With Reduced Trimethylamine-N-Oxide Level in Patients With Large-Artery Atherosclerotic Stroke or Transient Ischemic Attack	Yin, J	Journal Of The American Heart Association	2015	216	21.60
4	Gut Microbiota-Derived Short-Chain Fatty Acids Promote Poststroke Recovery in Aged Mice	Lee, J	Circulation Research	2020	160	32.00
5	Translocation and dissemination of commensal bacteria in post-stroke infection	Stanley, D	Nature Medicine	2016	154	17.11
6	Age-related changes in the gut microbiota influence systemic inflammation and stroke outcome	Spychala, MS	Annals of Neurology	2018	152	21.71
7	Transplantation of fecal microbiota rich in short chain fatty acids and butyric acid treat cerebral ischemic stroke by regulating gut microbiota	Chen RZ	Pharmacological Research	2019	147	24.50
8	Gut flora metabolism of phosphatidylcholine promotes cardiovascular disease	Wang ZN	Nature	2011	135	9.64
9	Gut Microbial Metabolite TMAO Enhances Platelet Hyperreactivity and Thrombosis Risk	Zhu WF	Cell	2016	134	14.89
10	Change of intestinal microbiota in cerebral ischemic stroke patients	Li N	BMC Microbiology	2019	133	22.17

To identify pivotal publications within the stroke-gut microbiota field that exhibit both sustained influence and contemporary relevance, we conducted a citation density analysis. This approach was employed to delineate the characteristics of literature with enduring impact and to systematically elucidate the evolution of research fronts and developmental trajectories. The articles by Singh et al. and Benakis et al., which possess not only the highest total citation counts (294 and 254, respectively) but also exceptional citation density indices (32.67 and 28.22, respectively), primarily investigate post-stroke neuroinflammation linked to microbiota dysbiosis and the immunomodulatory mechanisms of gut-resident γδ T cells. Since their publication in 2016, these two works have consistently attracted significant scholarly attention, establishing themselves as incontrovertible cornerstone and hotspot references in the field. It is noteworthy that, despite a marginally lower aggregate citation count, the publications by Lee et al. and Chen et al. also demonstrate relatively high citation densities (32.00 and 24.50, respectively). These studies converge on the therapeutic potential of gut microbiota-derived metabolites, specifically SCFAs. Their remarkably high annual citation rates signify that this line of inquiry represents one of the most rapidly advancing and promising research directions currently.

Based on the co-citation network, clustering and burst detection analyses were performed. The clustering network is shown in Figure 7B, with a modularity Q value of 0.9729 (>0.3000) and a weighted average silhouette coefficient value S = 0.9909 (>0.7000). The clustering network has a significant and reliable modular structure. The network includes seven clusters, each with a corresponding label. These clusters can be divided into three main topics: proteasome core biology, disease-related applied research, and drug development and methodology.

Figure 7C displays the top 25 references with the strongest burst detection. The reference with the highest burst intensity (intensity = 6.47) is “Intestinal microbiota metabolism of L-carnitine, a nutrient in red meat, promotes atherosclerosis”. This study reveals the complete mechanism by which L-carnitine in red meat is metabolized by the gut microbiota to produce trimethylamine N-oxide (TMAO), which directly promotes atherosclerosis. Given that atherosclerosis is the most common cause of ischemic stroke, this finding provides the most critical mechanistic evidence for the “diet-gut microbiota-host metabolism-stroke” axis. Additionally, four references have maintained citation heat up to 2023, with research focuses on the causal associations between gut microbiota and cardiovascular and cerebrovascular diseases, large-scale population cohort validation, and systematic evaluation methods. These studies emphasize the core role of using genetic methods such as Mendelian randomization to elucidate the causal relationship between gut microbiota and stroke risk, mapping population-level microbial characteristics through metagenomics, and integrating high-quality evidence in accordance with the PRISMA guidelines in advancing the field.

In summary, the core research focus within the field of post-stroke gut microbiota centers on dysbiosis, neuroinflammation, and the mechanistic underpinnings of the “brain-gut-immune axis”. Seminal studies, particularly those by Singh et al. and Benakis et al., have had a profound impact, establishing a crucial theoretical foundation for the entire field. More recently, the metabolism of short-chain fatty acids (SCFAs) and their therapeutic potential have emerged as a prominent new research frontier. The prevailing research trajectory is shifting from fundamental mechanistic exploration toward clinical translation and the development of intervention strategies. This evolution is providing critical insights for elucidating the pathological mechanisms underlying stroke-related gut microbiota dysbiosis and for advancing the development of precision treatment protocols.

### 3.8 Analysis of keywords

**Figure 8:**
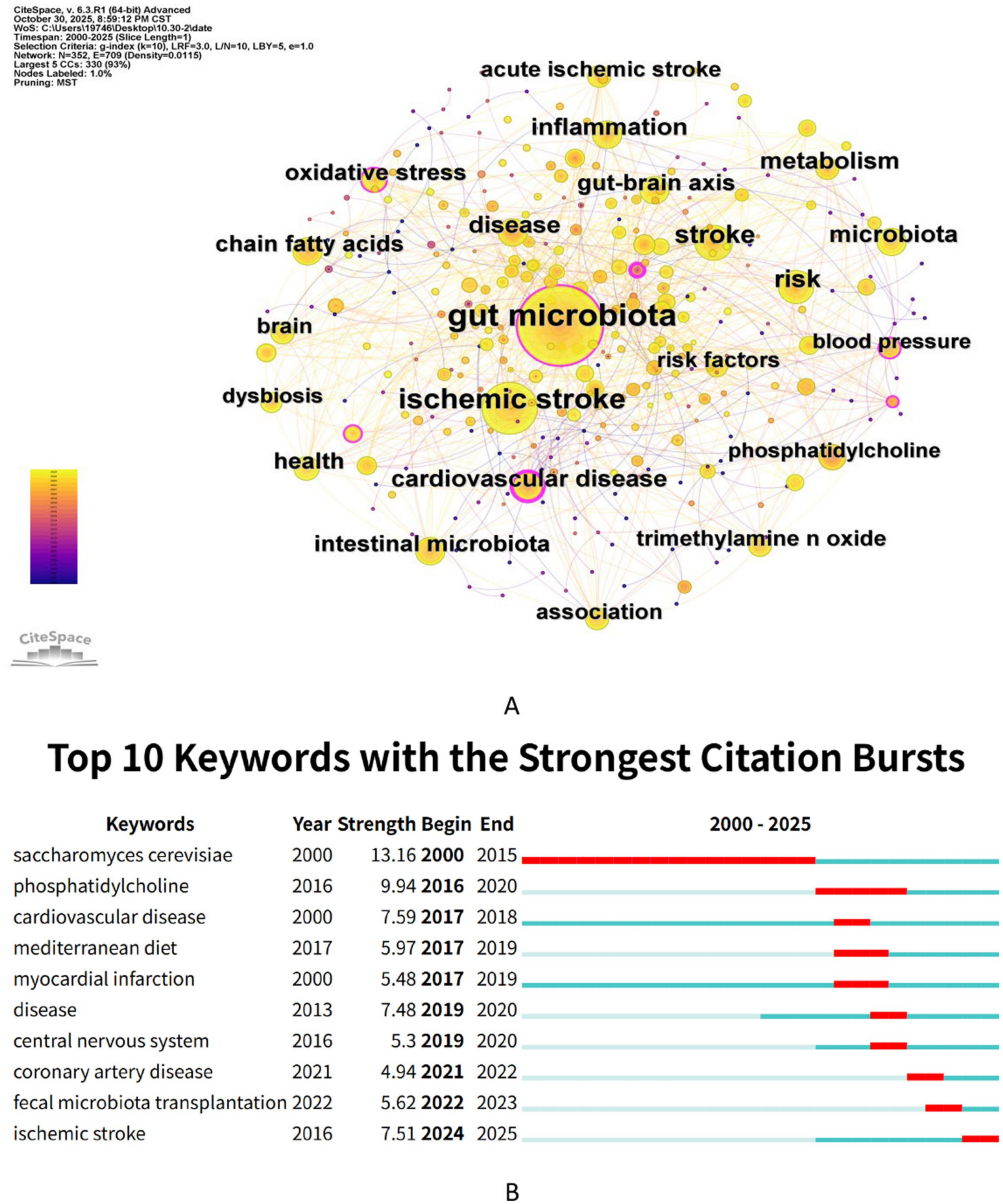
Co-occurrence and burst analysis of keywords. **(A)** Keyword co-occurrence network map. **(B)** Top 10 keywords with the strongest citation bursts.

Through co-occurrence analysis of keywords, the associations between keywords in stroke and gut microbiota research were identified. The co-occurrence network is shown in Figure 8A, and the top 20 high-frequency keywords are listed in Table 6. The keywords with the highest frequency of occurrence are “Gut microbiota” (490 times), “Stroke” (372 times), “Inflammation” (213 times), “Microbiota” (159 times), and “Risk” (127 times). Among them, “Gut microbiota” (0.87) and “Stroke” (0.79) have the highest betweenness centrality values, indicating that these two keywords are at the core of the entire research network and serve as important bridges connecting various fields of stroke and gut microbiota research. “Inflammation” (0.75), as the third highest betweenness centrality keyword, highlights the key connecting role of inflammatory mechanisms in this research domain.

**Table 6:** The top 20 most frequent keywords.

Rank	Keyword	Occurrence	Centrality
1	Gut microbiota	490	0.87
2	Stroke	372	0.79
3	Inflammation	213	0.75
4	Microbiota	159	0.66
5	Risk	127	0.70
6	Metabolism	115	0.65
7	Dysbiosis	104	0.51
8	Oxidative stress	99	0.59
9	Disease	94	0.69
10	Ischemic-stroke	94	0.67
11	Brain	92	0.61
12	Gut-brain axis	92	0.22
13	Intestinal microbiota	91	0.66
14	Atherosclerosis	88	0.42
15	Chain fatty-acids	88	0.70
16	Gut microbiome	86	0.30
17	Health	84	0.63
18	Probiotics	80	0.42
19	Microbiome	79	0.20
20	Association	73	0.58

Figure 8B reveals emerging trends in research through burst detection analysis. The top three keywords with the highest burst strength are “*Saccharomyces cerevisiae*” (strength = 13.16), “phosphatidylcholine” (strength = 9.94), and “central nervous system” (strength = 7.59). The keyword with the longest burst duration is “*Saccharomyces cerevisiae*,” lasting from 2000 to 2015. Meanwhile, “coronary artery disease”, “fecal microbiota transplantation” and “ischemic stroke” have remained active up to the present.

**Figure 9:**
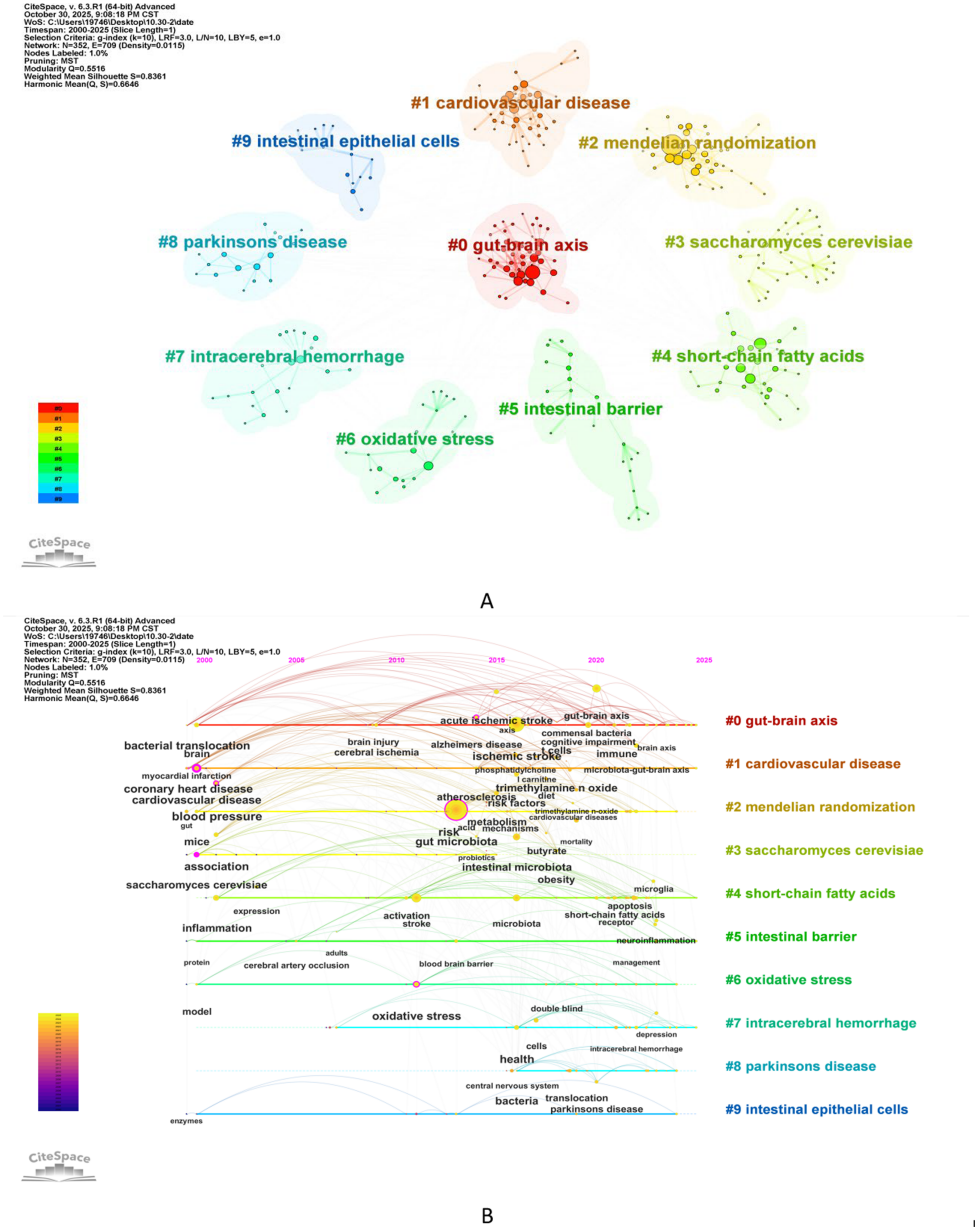
Cluster analysis of keywords. **(A)** Keywords clustering visualization map. **(B)** The timeline visualization map based on cluster analysis.

Figure 9A shows that keyword clustering analysis generated 10 clusters, with a modularity Q value of 0.5516 (>0.3000) and a weighted average silhouette value S = 0.8361 (>0.7000), confirming the stability of the network structure. The top three most active clusters are “#0 gut-brain axis”, “#1 cardiovascular disease”, and “#2 mendelian randomization”. The 10 clusters can be divided into three main research areas: the role of the gut-brain axis in stroke, and the study of gut microbiota-related metabolism and cardiovascular and cerebrovascular risks.

Figure 9B reveals the historical trajectory of research hotspots, the evolution of themes, and future trends through keyword timeline analysis. Early research (2000–2015) centered on “cardiovascular disease”, “oxidative stress”, and “gut microbiota” primarily focusing on the basic epidemiological associations and macro mechanisms between stroke and gut microbiota. Between 2015 and 2020, “short-chain fatty acids”, “intestinal barrier”, and “neuroinflammation” emerged as key topics, reflecting an increasing interest in the role of microbial metabolites and the intestinal barrier in the pathophysiology of stroke. Since 2020, keywords such as “Mendelian randomization”, “Parkinson’s disease”, and “intracerebral hemorrhage” have marked a deeper exploration of causal inference methods and the shared “gut-brain axis” mechanisms between stroke and other neurodegenerative diseases like Parkinson’s disease. This evolution clearly indicates that research in this field has shifted from initial phenomenological associations to exploring the complex interactions between multiple systems and diseases. This shift emphasizes the synergistic impact of gut microbiota dysbiosis and stroke on overall neurofunctional outcomes, such as cognitive impairment and reduced functional recovery. Notably, the persistent emphasis on “oxidative stress” and the “gut-brain axis” reaffirms their role as key pathological links, while terms like “probiotics” highlight the importance of microbial interventions in comprehensive disease management. Relevant studies have confirmed that probiotics have a protective effect on the neurological prognosis of stroke patients, with the key mechanism being the promotion of short-chain fatty acid (e.g., butyrate) production by specific strains in the gut, thereby improving intestinal barrier function, modulating neuroinflammation, and enhancing microglial homeostasis (Spychala et al., 2018). The core role of SCFAs lies in their protection of the neurovascular unit. Silva et al. pointed out that SCFAs, such as butyrate, can exert a core neuroprotective effect by enhancing blood–brain barrier function and inhibiting central inflammatory responses (Silva et al., 2020). Building on previous research, our findings indicate that microbiota-targeted strategies centered on SCFAs have broad application prospects in the prevention and treatment of stroke.

### 3.9 Cross-database validation of results

To evaluate the robustness of the research results based on WOSCC, we conducted equivalent searches in the Scopus and PubMed databases. Within the specified time range and inclusion criteria, we obtained 1,391 and 1,087 documents, respectively. Although absolute document numbers fluctuated due to differences in database coverage, the annual publication trends across the three databases were highly consistent, with Pearson correlation coefficients of 0.983 (WOSCC vs. Scopus) and 0.988 (WOSCC vs. PubMed), both of which were statistically significant (*p* < 0.001). The distribution of high-frequency keywords also showed significant overlap. For example, “gut microbiota,” “stroke,” “inflammation” and “microbiota” all ranked in the top 10 in all three datasets, indicating consistent research foci. The major contributing countries also remained consistent across different databases, with China and Japan still ranking first and second in publication output.

To further explore the characteristic differences among different literature databases, this study conducted a systematic comparative analysis of WOSCC, PubMed, and Scopus. In terms of keywords, after excluding the high-frequency core keywords common to all three databases, the unique keywords in the WOSCC database were more inclined to clinical feature terms (such as “risk,” “disease,” “metabolism”), reflecting its emphasis on translational medical research; the PubMed database contained a large number of research design-related terms (such as “humans,” “mice,” “aged”), reflecting the characteristics of basic medical research; it is worth noting that the unique keywords of the Scopus database included more refined technical concepts, such as “metabolomics” and “acute ischemic stroke”. Although these terms did not rank in the top 20 high-frequency keywords of WOSCC, they demonstrated Scopus’ extensive collection of cutting-edge technical terms and more precise disease classifications. This difference was also confirmed at the journal level: the number of unique journals in Scopus (19) was significantly higher than that of WOSCC (8) and PubMed (7), indicating its more comprehensive literature coverage. These findings not only revealed the complementary nature of different databases but also highlighted the importance of multi-database analysis for a comprehensive grasp of the research field.

## 4 Discussion

In this study, a bibliometric analysis of publications related to gut microbiota and stroke since 2000 was conducted using CiteSpace. By examining publication trends, co-citation networks, and keyword evolution, this analysis systematically revealed the research landscape, knowledge structure, and emerging hotspots within the field. Although the interaction between stroke and the gut microbiota has become a prominent research hotspot, the field remains at a critical juncture, transitioning from mechanistic exploration toward clinical translation.

### 4.1 High-cited literature and research trends with surging citations

Analysis of highly cited literature is an effective method for identifying the knowledge base of a research field. In the present study, the works of Singh et al. and Benakis et al. occupied central positions in the co-citation network, forming an important knowledge foundation in this field. These studies suggest that gut microbiota dysbiosis following stroke may activate inflammatory responses in the central nervous system by modulating immune mechanisms, particularly through the regulation of intestinal γδ T cells, thereby influencing stroke outcomes via the gut-brain axis. This theoretical framework has not only directed increasing research attention toward microbial metabolites and their interactions with the immune system, but has also highlighted SCFAs as an emerging research hotspot. Collectively, these findings have provided new mechanistic insights and identified potential clinical avenues for improving stroke prognosis through the targeted modulation of the gut microbiota (Sadler et al., 2020).

Citation burst analysis further revealed the dynamic evolution of research hotspots in this field. The present study found that several publications experienced a rapid increase in citations over a short period, indicating the growing depth of research on stroke and the gut microbiota. These articles predominantly focus on specific pathological mechanisms linking the gut microbiota to stroke, with particular emphasis on the role of short-chain fatty acids (SCFAs; Chen et al., 2019), the regulation of immune and inflammatory responses by gut microbiota, and their direct impact on brain injury and repair (Lee et al., 2020). Furthermore, preclinical and clinical studies investigating microbial interventions, such as probiotics, are garnering increasing attention. This trend signifies a deepening of the field, marking a progressive shift from fundamental mechanistic research toward clinical translation and application (Rahman et al., 2024).

Although the aforementioned studies have provided important insights into the relationship between gut microbiota and stroke, several critical issues remain to be addressed. First, the causal relationship between gut microbiota alterations and stroke has not yet been fully clarified. Most existing studies have reported associations between gut microbial dysbiosis and stroke occurrence or prognosis; however, it remains unclear whether specific microbial changes act as driving factors in the pathological process of stroke or represent secondary alterations resulting from disease progression. More rigorous causal inference will require advanced research approaches, such as germ-free animal transplantation experiments and prospective cohort studies. Second, multiple immune regulatory mechanisms may coexist, and their generalizability and specificity remain to be further elucidated. For example, the study by Benakis et al. highlighted the important role of the γδT cell pathway in gut–brain immune regulation, whereas the work by Singh et al. further suggested that various immune cell populations, including regulatory T cells, may also be involved. Future studies are needed to further elucidate the interactions among these immune pathways under different stroke subtypes and comorbid conditions, such as hypertension and diabetes, as well as to explore the potential existence of individualized immune regulatory patterns. Third, translating mechanistic insights into effective clinical interventions remains a major challenge. Although highly cited studies suggest the therapeutic potential of targeting the gut microbiota, simple probiotic supplementation or fecal microbiota transplantation still faces several obstacles in clinical practice, including colonization resistance, long-term safety concerns, and inter-individual variability in treatment responses. Therefore, future research should focus on developing more precise microbiota-based intervention strategies based on a deeper understanding of the underlying mechanisms, in order to facilitate further clinical translation in this field.

### 4.2 Research hotspots: gut microbiota, SCFAs, and gut-brain axis

Keyword co-occurrence and clustering analysis clearly reveals the core knowledge structure and research hotspots within the field of stroke and gut microbiota. The terms “gut microbiota” “stroke” and “inflammation” exhibited the highest betweenness centrality indicating that research in this domain primarily focuses on the interrelationships among gut microbiota alterations stroke pathogenesis and progression and inflammatory responses. Concurrently “short-chain fatty acids” and the “gut–brain axis”—Identified as keywords exhibiting both high frequency and strong citation bursts—Suggest that microbial metabolites and gut-brain axis-related mechanisms have emerged as important research frontiers in recent years. Among these SCFAs are considered key mediators linking the gut microbiota to the pathological processes of stroke and have therefore garnered increasing attention from the research community. SCFAs especially butyrate, acetate, and propionate are considered metabolites with important biological functions produced by the gut microbiota (Srivastav et al., 2019) and they play a central role in the pathophysiological process of stroke. Keyword analysis in this study reveals that SCFA-related mechanisms have emerged as a significant research hotspot with current investigations focusing on three primary areas: The regulation of neuroinflammation (Benakis et al., 2016) the maintenance of gut barrier and blood–brain barrier integrity (Spychala et al., 2018) and the direct modulation of microglial function (Lee et al., 2020). Accumulating evidence indicates that SCFAs can cross the blood–brain barrier and exert neuroprotective effects thereby promoting neurological functional recovery. These benefits are achieved at least in part by alleviating central and systemic post-stroke inflammation through mechanisms that include inhibiting the release of pro-inflammatory cytokines and enhancing the expression of tight junction proteins (Cao et al., 2025; Pu et al., 2025). Furthermore recent bioinformatics-based studies have elucidated the mechanistic associations between SCFA-related genes and stroke-related pathways providing additional support for the pivotal role of gut-derived metabolites in modulating the immune response to stroke (Ding et al., 2024).

### 4.3 Mechanism of intestinal flora in stroke

Building upon the research hotspots identified above, this section integrates existing evidence to provide a comprehensive analysis of the potential mechanisms through which the gut microbiota influences stroke. Current evidence suggests that the gut microbiota may influence the pathogenesis and progression of stroke through multiple interconnected pathways. These primarily involve microbial metabolites, immune regulation, and the integrity of the gut–brain barrier, all of which are closely linked to neuronal injury and subsequent functional recovery.

#### 4.3.1 Dysbiosis of intestinal flora and stroke risk

Keyword co-occurrence analysis in this study identified “inflammation” and “gut microbiota” as major research themes suggesting that gut microbiota dysbiosis and inflammatory responses may play pivotal roles in the pathophysiology of stroke. Previous studies have demonstrated that stroke patients typically exhibit a reduction in beneficial bacterial genera (e.g., Lactobacillus and Bifidobacterium) alongside an increase in potentially pro-inflammatory taxa (e.g., Enterobacteriaceae and Bacteroidaceae) accompanied by an overall decline in gut microbial diversity (Yamashiro et al., 2017). This microbial imbalance may increase stroke risk through several interrelated pathways. First gut microbiota dysbiosis is frequently associated with an overgrowth of potentially pro-inflammatory gram-negative bacteria. These bacteria release lipopolysaccharides (LPS) into the bloodstream which can trigger systemic inflammatory responses. This cascade may lead to vascular endothelial damage and thrombosis thereby elevating the risk of stroke (Zhu et al., 2016; Brown and Hazen, 2018). Second microbial dysbiosis can disrupt the normal metabolism of fatty acids bile acids and tryptophan. This disruption may enhance the production of oxidative stress-related and neurotoxic metabolites ultimately exacerbating cerebrovascular and neuronal damage (Agirman et al., 2021). Furthermore the gut microbiota plays a crucial role in regulating host immune homeostasis. Gut microbiota dysbiosis can disrupt this balance leading to a reduction in regulatory T cells (Tregs) and an expansion of pro-inflammatory Th17 cells. This imbalance can trigger inflammatory cascade reactions thereby creating a permissive environment for cerebrovascular injury (Benakis et al., 2016). Further animal studies have demonstrated that modulating the gut microbiota can partially reverse inflammation metabolic and immune abnormalities caused by dysbiosis thereby reducing the risk of stroke and improving post-stroke neurological recovery (Yin et al., 2015).

#### 4.3.2 Mechanism and potential applications of SCFAs in ischemic brain injury

Keyword analysis in this study revealed that SCFAs exhibited both high frequency and strong citation burst intensity indicating that this topic has emerged as a significant research direction within the field. SCFAs are key metabolites generated through the fermentation of dietary fiber by the gut microbiota. They are increasingly recognized as critical mediators in the regulation of post-stroke inflammatory responses and neuronal injury. In terms of immune regulation SCFAs promote the differentiation of regulatory T cells (Treg) by activating G protein-coupled receptors (GPR41/GPR43) and inhibit the release of pro-inflammatory cytokines (such as IL-1β, IL-6, TNF-*α*) thereby reducing microglia-mediated neuroinflammatory responses (Haghikia et al., 2015). In terms of blood–brain barrier maintenance SCFAs effectively enhance the integrity of the blood–brain barrier and reduce its permeability by upregulating the expression of tight junction proteins (such as Occludin and Claudin-5) preventing the entry of peripheral inflammatory mediators and other harmful substances into the brain parenchyma (Braniste et al., 2014; Liu et al., 2017). In terms of neuroprotection and repair SCFAs increase neuronal survival rates and promote post-stroke neurological function recovery by inhibiting histone deacetylase and activating the Nrf2/Keap1 signaling pathway to regulate intracellular oxidative stress responses (Long et al., 2022; Kalyanaraman et al., 2024). Previous studies have shown that SCFAs supplementation or improvement of the gut microbiota can significantly reduce cerebral infarct area and improve motor function scores indicating its potential application value in stroke rehabilitation (Chen et al., 2019).

#### 4.3.3 Functions of other intestinal microbial metabolites and signaling pathways in stroke

While the bibliometric findings of this study predominantly highlight mechanisms centered on the “gut microbiota-inflammatory response-SCFAs” axis, recent basic and clinical evidence indicates that the gut microbiota can also influence the progression of ischemic stroke through a broader array of metabolites and signaling pathways. Based on their functional effects, these metabolites can be broadly categorized into pro-damaging factors (e.g., trimethylamine N-oxide [TMAO] and bacterial lipopolysaccharides) and protective factors (e.g., tryptophan metabolites and secondary bile acids), which influence stroke outcomes through pro-inflammatory and neuroprotective pathways, respectively. Specifically, TMAO is a metabolite generated by the gut microbiota’s breakdown of dietary precursors such as choline and carnitine. It can promote platelet activation and endothelial dysfunction, induce vascular inflammatory responses, and increase the risk and severity of ischemic stroke by accelerating the formation of atherosclerosis and thrombosis (Dolkar et al., 2024). Bacterial lipopolysaccharides can enter the body through the circulation when intestinal barrier function is impaired. They bind to TLR4 receptors on the surface of cerebral vascular endothelial and immune cells, activate the NF-κB signaling pathway, induce the release of pro-inflammatory cytokine cascades, and exacerbate inflammation and blood–brain barrier disruption after cerebral ischemia (Jiang et al., 2025). In contrast, certain gut-derived metabolites exert neuroprotective effects. For instance, tryptophan metabolites—primarily indoles and their derivatives—can regulate the differentiation of peripheral immune cells and microglial activity by activating the aryl hydrocarbon receptor (AhR). This activation inhibits the release of pro-inflammatory cytokines, thereby attenuating neuroinflammation and neuronal damage in the brain (Owe-Larsson et al., 2025; Qin et al., 2025). Secondary bile acids are generated by the gut microbiota through the biotransformation of primary bile acids. These metabolites can activate the FXR and TGR5 signaling pathways, improve lipid and energy metabolism, inhibit neuroinflammatory responses, and promote neuronal survival. Through these combined mechanisms, they offer potential protective effects against cerebral ischemia (Romero-Ramírez and Mey, 2024).

### 4.4 Differences in gut microbiota profiles between ischemic and hemorrhagic stroke

The bibliometric findings of this study reveal a disparity in the developmental trajectory of research on ischemic versus hemorrhagic stroke within the gut microbiota field. Based on keyword distribution, “ischemic stroke” emerged as a persistently active, high-frequency keyword throughout the study period. Keyword burst detection (Figure 8B) further revealed that this term has remained continuously active since its first appearance, indicating that the interplay between ischemic stroke and the gut microbiota has consistently been a central research focus within the field. This finding aligns with the epidemiological prominence of ischemic stroke and reflects its dominant role in both clinical research and mechanistic investigations. In contrast, research on hemorrhagic stroke is gradually gaining momentum. Timeline analysis of keywords (Figure 9B) indicates that “intracerebral hemorrhage” has emerged as a research hotspot since approximately 2020, suggesting that the association between hemorrhagic stroke and the gut microbiota is beginning to attract scholarly attention. Analysis of highly cited literature (Table 5; Figure 7) further corroborates this trend. The most influential core publications in this domain primarily focus on immune regulatory mechanisms linking post-ischemic stroke gut microbiota dysbiosis with neuroinflammation. By comparison, highly influential studies focusing on hemorrhagic stroke have not yet coalesced into a distinct knowledge cluster. Multi-database validation analysis revealed that more specific disease classification terms, such as “acute ischemic stroke”, appeared among the keywords in the Scopus database, indicating a growing focus on ischemic stroke subtype research within the field.

### 4.5 Limitations

This study has several limitations that should be acknowledged. First, although multi-database validation was performed to enhance the robustness of the findings, the primary bibliometric analysis was conducted using only the WoSCC database. Data from Scopus and PubMed were used solely for cross-validation purposes and were not integrated into the core analysis. Consequently, some unique publications indexed exclusively in these databases may have been omitted. Second, recently published high-quality articles may not have had sufficient time to accumulate citations. This time lag means that their full academic impact may not yet be fully reflected in the present analysis. Finally, the restriction to English-language publications may introduce selection bias, potentially overlooking significant contributions from non-English literature. Future studies should aim to expand data sources to include multilingual literature and incorporate longitudinal data analysis. Such approaches would enhance the global representativeness and analytical depth of the findings.

### 4.6 Future directions

Based on the bibliometric findings of this study, future research on the relationship between the gut microbiota and stroke could be advanced in several key directions. Given that research on ischemic stroke has become a major focus whereas investigations into hemorrhagic stroke remain in an early stage, future studies should prioritize stratification by stroke subtype. Specifically, systematic comparative analyses of gut microbial composition and the underlying pathological mechanisms between these two subtypes are warranted. Furthermore, more refined analyses of etiological subtypes within ischemic stroke could provide a stronger theoretical foundation for the development of precision interventions. Concurrently, multi-omics approaches—including metabolomics, metagenomics, and proteomics—should be integrated to construct comprehensive microbiota–host interaction networks. This will facilitate a deeper understanding of the mechanistic roles played by microbial metabolites in the pathogenesis and progression of stroke. Keyword analysis indicates that Mendelian randomization has emerged as a research hotspot in recent years, suggesting that such genetic approaches should be more widely applied to infer potential causal relationships between specific microbial taxa and distinct stroke subtypes. Furthermore, well-designed multicenter prospective cohort studies are needed to validate the clinical utility of microbiota-related biomarkers for predicting stroke risk and assessing patient prognosis. Keyword burst detection further reveals sustained and growing interest in intervention strategies, particularly fecal microbiota transplantation. Consequently, future randomized controlled trials spanning different stroke subtypes are warranted to rigorously evaluate the safety and efficacy of such interventions, thereby facilitating the translation of fundamental research findings into clinical practice.

## 5 Conclusion

This study, employing bibliometric methods, systematically analyzed the research landscape, knowledge structure, and emerging trends in the field of stroke and gut microbiota over the period from 2000 to 2025. The analysis reveals that the field has rapidly evolved from an emerging theoretical framework into a vibrant and highly interdisciplinary domain, characterized by significant cross-disciplinary integration. Current research hotspots are highly concentrated on the pivotal roles of gut microbiota dysbiosis, neuroinflammation, and key metabolites, particularly short-chain fatty acids (SCFAs), within the “gut-brain axis” framework. Future research priorities will center on transitioning from mechanistic exploration to clinical translation, with a particular emphasis on developing personalized, microbiome-based diagnostic and therapeutic strategies. Such advancements are poised to offer novel avenues for the prevention and treatment of stroke.

## Data Availability

The original contributions presented in the study are included in the article/Supplementary material, further inquiries can be directed to the corresponding authors.

## Footnotes

1
https://jcr.clarivate.com/
